# Web-based cognitive bias modification for problem drinkers: protocol of a randomised controlled trial with a 2x2x2 factorial design

**DOI:** 10.1186/1471-2458-13-674

**Published:** 2013-07-20

**Authors:** Denise S van Deursen, Elske Salemink, Filip Smit, Jeannet Kramer, Reinout W Wiers

**Affiliations:** 1 Addiction Development and Psychopathology (ADAPT)-lab, Department of Psychology, University of Amsterdam, Weesperplein 4, 1018 XA Amsterdam, The Netherlands; 2 Trimbos Institute, Netherlands Institute of Mental Health and Addiction, PO Box 725, 3500 AS Utrecht, The Netherlands; 3 Department of Epidemiology and Biostatistics, EMGO Institute for Health and Care Research, VU University Medical Centre, Van der Boechorststraat 1, 1081 BT Amsterdam, The Netherlands

**Keywords:** Problem drinking, Alcohol, Cognitive bias modification, Implicit cognition, E-health intervention

## Abstract

**Background:**

The automatic tendency to attend to, positively evaluate and approach alcohol related stimuli has been found to play a causal role in problematic alcohol use and can be retrained by computerised Cognitive Bias Modification (CBM). In spite of CBMs potential as an internet intervention, little is known about the efficacy of web-based CBM. The study described in this protocol will test the effectiveness of web-based CBM in a double blind randomised controlled trial with a 2 (attention bias retraining: real versus placebo) x 2 (alcohol/no-go training: real versus placebo) x 2 (approach bias retraining: real versus placebo) factorial design.

**Methods/design:**

The effectiveness of 12 sessions of CBM will be examined among problem drinkers aged 18–65 who are randomly assigned to one of the eight CBM conditions, after completing two modules of a validated cognitive behavioural intervention, *DrinkingLess*. The primary outcome measure is the change in alcohol use. It is expected that, for each of the CBM interventions, participants in the real CBM conditions will show a greater decrease in alcohol use than participants in the placebo conditions. Secondary outcome measures include the percentage of participants drinking within the limits for sensible drinking. Possible mediating (change in automatic biases) and moderating (working memory, inhibition) factors will be examined, as will the comparative cost-effectiveness of the various CBM strategies.

**Discussion:**

This study will be the first to test the relative efficacy of various web-based CBM strategies in problem drinkers. If proven effective, CBM could be implemented as a low-cost, low-threshold adjuvant to CBT-based online interventions for problem drinkers.

**Trial registration:**

Netherlands Trial register: NTR3875.

## Background

Problematic alcohol use is highly prevalent and is associated with substantial costs for both the individual and society [1]. This calls for (cost-) effective interventions targeting the early stages of the disease process. Web-based interventions might meet these demands, by providing effective, low-cost interventions aimed at reducing problem drinking [2,3]. That said, the effectiveness of web-based interventions might be limited by their traditional focus on altering conscious (i.e. intentional, controlled) aspects of information processing, while many recent theories of addiction underscore the complementary role of more automatic, less intentional processes in the aetiology and maintenance of substance use problems [4-6]. According to dual process theories, repeated alcohol use can cause automatic (i.e. fast, less conscious) biases in information processing: alcohol cues selectively capture attention (attentional bias), activate positive associations in memory (memory bias), and elicit automatic approach tendencies (approach bias). These biases have been found to predict alcohol use [7,8], especially in people with low working memory [9,10], or poor response inhibition [11,12]. Importantly, these biases are largely unaffected by interventions aimed at altering conscious information processing [13,14], suggesting that interventions for problem drinkers will be more effective when both controlled and automatic processes are addressed.

During the past decade, a set of computerised training programs has been developed aimed at altering automatic cognitive biases, collectively called Cognitive Bias Modification (CBM). CBM has been found effective at altering attentional bias [15-18], memory bias [19,20], and approach bias [21-23], often with associated impacts on alcohol use. While CBM was first employed to test the causal role of automatic processes in alcohol use, researchers have now begun to examine CBM’s relevance for therapeutic intervention. Three studies have investigated the effects of CBM as an add on to face-to-face treatment, showing favourable effects on treatment duration [16] and relapse [21,23] among patients with alcohol dependence. While the promising effects of ‘offline’ CBM as a supplement to traditional interventions point to the possible value of ‘online’ CBM as an addition to current web-based interventions, little is known about the effectiveness of web-based CBM (but see [24] for an example of online working memory training among problem drinkers). This is surprising given the fact that CBM procedures were all developed as computerised tasks that require little explanation, thereby facilitating their suitability as web-based self-help interventions. Furthermore, in the field addiction, no studies have yet been published on the effects of combining different CBM interventions, though this is likely to maximize its efficacy.

### Aims and hypotheses

The aim of the current study is to investigate the effectiveness of three online CBM interventions: attentional bias retraining, alcohol/no-go training (targeting memory bias), and approach bias retraining. Problem drinkers receive 12 sessions of either the active or the placebo version of all three training programs, after completing the first two modules of an evidence-based online cognitive behavioural therapy (CBT) based intervention, *DrinkingLess*[25]*,* consisting of personalised feedback on one’s alcohol use and setting personal goals related to alcohol use, in order to increase motivation to change. The main goal of the study is to test the added effects of the CBM interventions on alcohol use at 3 months after the intervention, with changes in the number of alcoholic drinks consumed in the past two weeks as the primary outcome measure. It is expected that, for each of the CBM interventions, participants in the intervention conditions will show a greater decrease in weekly alcohol use than participants in the placebo conditions. Furthermore, each CBM intervention is hypothesised to decrease or reverse the specific bias it trains and these changes are expected to mediate the effects on alcohol use. Spill-over effects of each specific CBM intervention to other biases will be explored, as will the effects of the joint exposure to the various combinations of CBM interventions. In order to answer the question of “what works for *whom*?”, possible moderators will be examined. In line with dual process models, it is expected that participants with strong automatic biases and/or low working memory capacity and inhibitory control will benefit more from CBM than participants with weaker biases and/or stronger executive functions (as was found for CBM both in addiction [21] and in anxiety [26,27]). A better understanding of the relative contribution of each CBM version on treatment outcome is also expected to come from examining the cost-effectiveness of CBM interventions. Knowledge about the most effective CBM version(s) may have important implications for making choices in the implementation of CBM in health care.

## Methods/design

### Trial design

**Figure 1:**
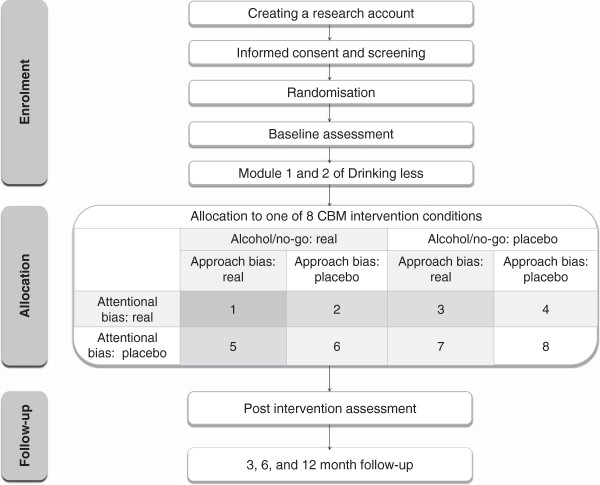
Participant flowchart.

**Figure 2:**
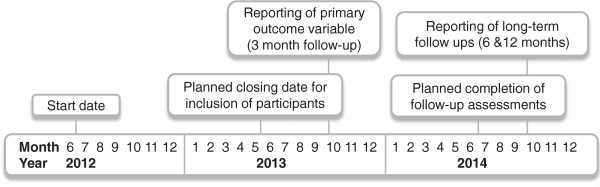
Expected timeline for completion and reporting.

The (cost) effectiveness of attentional bias retraining, alcohol/no-go training, and approach bias retraining will be studied in a 2×2×2 factorial design. This will allow us to explore possible additive or multiplicative effects of different combinations of CBM, as well as the related question to what extent retraining one bias will produce changes in other automatic biases; issues which not only have clinical relevance, but also important theoretical implications (see for example [28,29]). Participants will receive either the active or the placebo version of all three CBM interventions, amounting to eight conditions (see Figure 1). Prior to the training, participants will complete the first two modules of the *DrinkingLess* program [25]*.* Intervention effects will be tested directly after the intervention and 3, 6 and 12 months later. The expected timeline for trial completion and reporting the results is presented in Figure 2.

### Participants and procedure

To ensure the ecological validity of the sample, participants will be recruited through sources that are comparable to how internet interventions are generally promoted in the Netherlands: the *DrinkingLess* website, websites related to alcohol abuse, newspaper articles, a television documentary on CBM for addiction, the website of the ADAPT lab, and word of mouth communication with existing participants. Interested participants are invited to create an account on the intervention website (http://www.test.uva.nl/toptrainingen), after which they read the information letter, a copy of which is automatically sent to them by e-mail. Participants who sign the online consent form then complete a brief screening for eligibility. Participants will be included if they: (a) have an Alcohol Use Disorders Identification Test (AUDIT [30]) score of 8 or above (b) have drunk more than 21 drinks (men) or 14 drinks (women) a week in the past two weeks, as assessed with the Timeline-Follow-Back (TLFB) method (c) are between 18–64 years old (d) have (almost) daily internet access and (e) do not receive professional treatment for problem drinking at the start of the study. Participants with an AUDIT score of >19 receive a message warning them that abrupt abstaining can have mental and physical consequences, and advising them to contact their General Practitioner in case of withdrawal symptoms. Eligible participants then complete the baseline assessment and two modules of *DrinkingLess*. The next day, they are invited to complete the first CBM training session. Participants have three days to complete each session, allowing them to complete the 12 training sessions in about 5 weeks. One day after the last training session, participants are invited to complete the post-intervention assessment. Follow-up assessments are conducted at three, six and twelve months after the intervention. The study has been approved by the Ethics Committee of the psychology department of the University of Amsterdam and registered at the Netherlands Trial register (NTR3875).

### Interventions

#### DrinkingLess

*DrinkingLess* is an evidence-based cognitive behavioural internet intervention [25]. Since most visitors of the *DrinkingLess* website were found to complete the first two out of four modules of the intervention [31], and these modules were also deemed most important to prepare participants for the CBM interventions, only these modules are offered in the current study. The modules include personalised feedback on alcohol use and its consequences, and setting personal goals related to alcohol use (see [25,31] for more information).

#### CBM interventions

Each CBM session consists of three tasks: attentional bias retraining, alcohol/no-go training, and approach bias retraining. Each task consists of three phases: a practice block, an assessment block (48 trials), and a CBM block (120 trials). The purpose of the assessment block is to measure the strength of the bias at the start of every session, to examine whether biases decrease as a result of CBM training. During the CBM block, participants will receive either the experimental or the placebo version of the specific CBM version. A large set of pictures of alcoholic drinks, non-alcohol drinks and neutral objects was created specifically for this study, the validity of which is currently studied in our lab. Each trial starts with a fixation cross which is presented in the middle of the screen for 500 ms. The inter-trial interval is 500 ms. Since some varieties of CBM have been perceived as boring and participants have trouble seeing how CBM could help them reduce their problems [32], special attention was given to the treatment rationale. Furthermore, a game-like system of earning points was included in order to keep participants motivated to respond accurately and quickly and to complete all CBM sessions.

#### Attentional bias retraining

**Figure 3:**
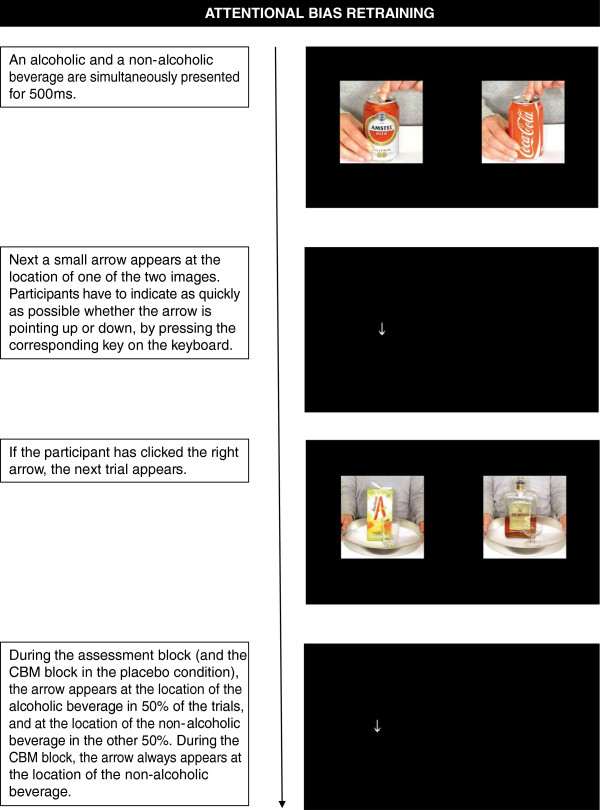
Example of a trial in the attentional bias retraining.

Attentional bias is assessed and trained using the visual probe task [16,33,34]. In this task, a picture of an alcoholic and a picture of a non-alcohol beverage are presented next to each other on the screen for 500 ms. When the pictures disappear, a small arrow pointing up or down appears at the location of one of the pictures. Participants are instructed to respond to the direction of the arrow, by pressing the corresponding arrow key on the keyboard. In the assessment block, the arrow replaces the picture of the alcoholic beverage (alcohol trials) and the picture of the non-alcoholic beverage (non-alcohol trials) equally often. Attentional bias for alcohol is computed by subtracting response times (RTs) on alcohol trials from those on non-alcohol trials. In the CBM block, participants in the experimental condition will be trained to direct their attention away from alcoholic beverages towards non-alcoholic beverages, by exposing them only to non-alcohol trials, while participants in the placebo condition receive 50% alcohol and 50% non-alcohol trials (as in the assessment block). For an example of a trial in the attentional bias retraining, see Figure 3.

#### Alcohol/no-go training

**Figure 4:**
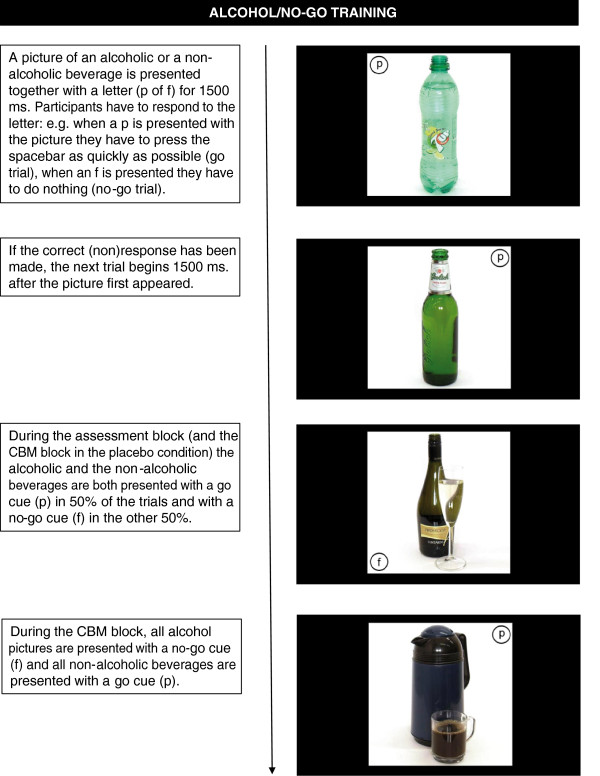
Example of a trial in the alcohol/no-go training.

Alcohol related memory bias (positive associations with alcohol) is measured and trained using the alcohol/no-go task [11]. In this task, a picture of an alcoholic or non-alcoholic beverage is presented for 1500 ms, together with a go (i.e. the letter ‘p’) or no-go cue (‘f’) which is displayed randomly in one of four corners of the picture. Participants are instructed to respond to the presented letter, by pressing the spacebar as quickly as possible when they see the letter ‘p’, and doing nothing (wait until picture disappears) when they see the letter ‘f’. The combination of the letter and the response (p = press and f = inhibit, versus f = press and p = inhibit) is counterbalanced across participants. In the assessment block, the pictures of alcoholic and non-alcoholic beverages are presented equally often with a go and with a non-go cue. Alcohol/go bias is computed by subtracting RT’s on alcohol/go trials from those on non-alcohol/go trials. In addition, the number of errors (incorrect go responses) can be compared between alcohol and non-alcohol no-go trials. In the CBM block, participants in the experimental condition will be trained to inhibit their response to alcohol, by exposing them only to alcohol/no-go trials and non-alcohol/go trials, while for participants in the placebo condition there is no relation between the content of the picture and the required response. For an example of a trial in the alcohol/no-go training bias retraining, see Figure 4.

#### Approach bias retraining

**Figure 5:**
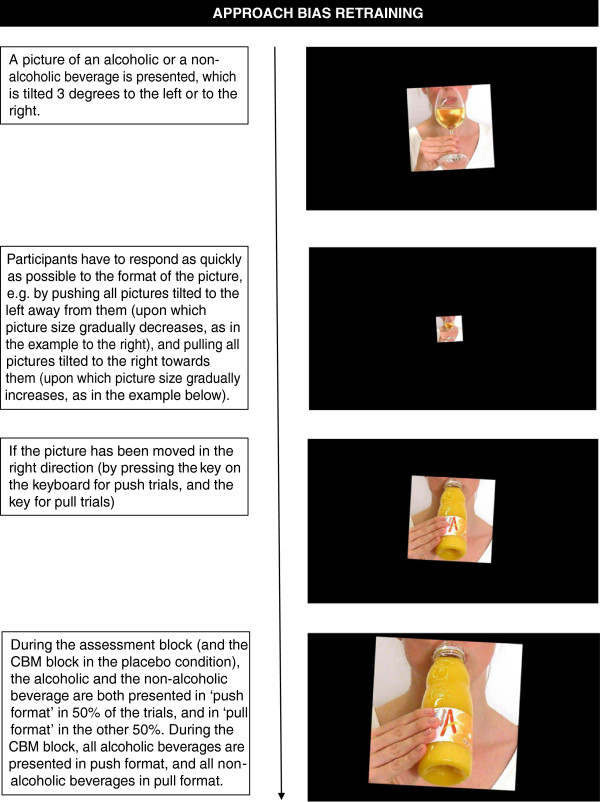
Example of a trial in the approach bias retraining.

Approach bias is measured and trained with the modified Approach-Avoidance Task (AAT [22]). In this intervention, a picture of an alcoholic or non-alcoholic beverage is presented, which is tilted 3 degrees to the left or right. Participants are instructed to respond to the format of the picture, by pushing all pictures tilted to the right away from them, and pulling all pictures tilted to the left towards them. The combination of the format of the picture and the response (left = push and right = pull, versus left = pull and right = push) is counterbalanced across participants. Picture size gradually increases when the pull-key is pressed, while it decreases when the push-key is pressed. In the assessment block, the pictures of alcoholic and non-alcoholic beverages are presented equally often in push and in pull format. Approach bias for alcohol is computed by comparing RTs for push, pull, alcohol and non-alcohol trials ((alcohol/push - alcohol/pull) - (non-alcohol/push - non-alcohol/pull)). In the CBM block, participants in the experimental condition will be trained to avoid alcohol, by exposing them only to alcohol/push and non-alcohol/pull trials, while for participants in the placebo condition there is no relation between the content of the picture and the required response. For an example of a trial in the alcohol/no-go training bias retraining, see Figure 5.

### Baseline measures

Eligibility for the study will be assessed with the TLFB [35] and the AUDIT [30]. Lifetime alcohol and drug use and use during the past month is assessed, as well as history of alcohol use problems (alcohol scales of the European Addiction Severity Index, EuropASI [36]). Participants indicating they smoked during the past month will fill out the Fagerström Test for Nicotine Dependence (FTND [37]). Motivation to change alcohol use is measured with the Readiness to Change Questionnaire (RCQ [38]). Symptoms of depression and anxiety are measured with the Beck Depression Inventory-II (BDI-II [39]), and the Spielberger State-Trait Anxiety Inventory (STAI [40]), respectively. High trait sensitivity, which is correlated with genes related to susceptibility to both positive and negative environmental influences [41,42], is measured as a possible predictor of the changeability of automatic biases (using a short version of the High Sensitive Personality Child Questionnaire (HSC [43]).

A computerised version of the Self-Ordered Pointing Task (SOPT [44] is used to assess working memory capacity. In this task, participants are presented with a grid of pictures of concrete objects, and are instructed to click on each picture with the computer mouse. There are two rules: one can only click each picture once, and one cannot click on the same location twice in a row. The task starts with a practice block of 4 pictures, and proceeds with 5 consecutive test blocks with 6, 8, 10, 12, and 12 pictures. The outcome is the correct number of unique pictures selected during the test blocks.

**Figure 6:**
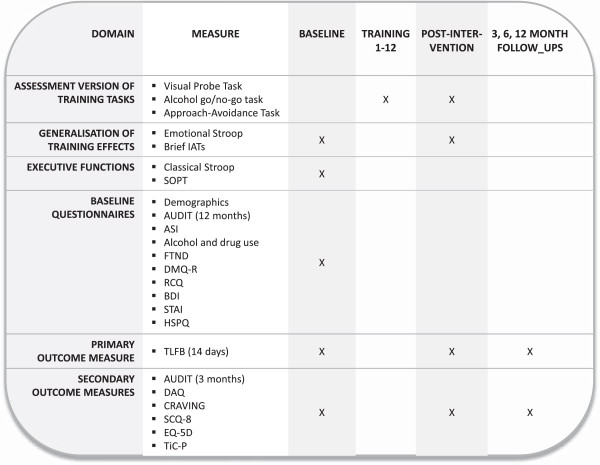
Measurement instruments.

The classical [45] and alcohol [46] Stroop are used to assess response inhibition and attentional bias, respectively. The task starts with a practice block, in which participants have to learn the correct key-colour combination. In the next block (alcohol Stroop), participants have to identify the colour of 7 alcohol-related words (e.g. wine, whiskey), and 7 matched neutral words (office supplies). Each word is presented in four colours, resulting in 56 trials. The final block consists of a classical Stroop task of 56 trials, with 8 congruent trials (red in red), 24 incongruent trials (red in green), and 24 neutral trials (%%% in green). An overview of all measurement instruments and assessments is presented in Figure 6.

### Primary and secondary outcome measures

The main outcome measure, the change in number of drinks consumed in the past 14 days three months after the intervention compared to baseline, will be assessed using the TLFB method [35]. Secondary outcome measures are: changes in automatic cognitive biases as assessed with the attentional bias, alcohol/no-go and approach bias tasks described above. Generalisation effects of attentional bias and approach bias training to other measures will be assessed with the alcohol Stroop task (described above) and the brief Implicit Association Task (bIAT, [47,48]), which is used to measure the strength of both approach associations and positive (valence) associations with alcohol. In the bIAT, participants are required to decide whether or not the exemplar word in the middle of the screen belongs to one or two focal categories at the top of the screen, by pushing the designated ‘yes’ or ‘no’ buttons (‘e’or ‘i’) on the keyboard. As in the study of Menatti et al. [47], the typical 7 block structure of the IAT was maintained, but with fewer trials per block. In the first block (12 trials) names of alcoholic (wine, beer, vodka) or non-alcoholic drinks (coke, water, juice) have to be classified as either belonging to the category ‘alcohol’ or not. In the second block (12 trials) exemplars have to be classified as either pleasant (funny, cheerful, sociable) or not (nauseous, sad, tired). In the third block (12 trials) and fourth (24 trials), the target (alcohol) and attribute (pleasant) categories are combined. In the fifth block (12 trials), the reversed attribute category, unpleasant, is practiced and then combined with the target category (alcohol) in the sixth (12 trials) and seventh block (24 trials). The approach bIAT follows the same structure, with the attributes ‘approach’ (grab, touch, take) ‘avoid’ (let go, avert, ignore). The order of the two versions of the bIAT, as well as the order of the combined blocks within the bIAT, and the contingency between the response (yes/no) and the buttons (i/e) is counterbalanced across participants. The outcome measure is the standardised difference in latencies (*D* score*)* between the different combined blocks.

Other secondary measures are: the percentage of participants drinking within the limits for sensible drinking [49]: a maximum of 21 standard units of alcohol per week for men, and 14 units a week for women) and binge drinking (drinking >5 glasses on one day), both measured with the TLFB [35]. Alcohol-related problems will be assessed with the AUDIT [30], which was adapted to a three month version in order to enhance its sensitivity to changes within the follow-up period of the study. Craving will be measured with the Desires for Alcohol Questionnaire (DAQ [50]), and by asking participants how much they want to drink each of three pictured alcoholic and non-alcohol beverages at this moment on a 10 point scale. Self-efficacy will be assessed with the Brief Situational Confidence Questionnaire (BSCQ [51]), in which participants are asked to indicate on a visual analogue scale how much confidence they have that they will not drink heavily in eight specific situations. Quality of life will be measured with the EQ-5D [52]. The economic costs stemming from health care uptake and productivity losses associated with problematic alcohol use will be assessed with the Trimbos/iMTA Questionnaire for Costs associated with Psychiatric illness (TiC-P [53]).

### Sample size

**Figure 7:**
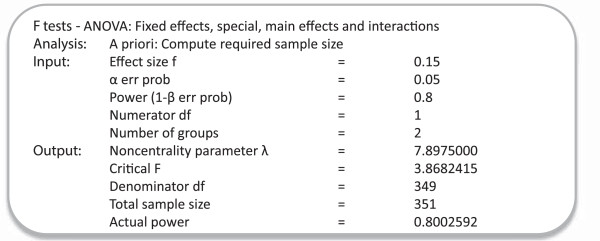
Sample size calculation using G*Power 3.1.5.

G*Power 3.1.5 [54] was used to calculate the sample size for a power of 0.8 for each main effect or interaction (all Numerator *df* = 1), at an alpha of 0.05. Based on a meta-analysis of internet self-help interventions for problem drinkers a medium effect size (*f* = 0.25) would be expected [2], but the effect size may be reduced by the active control conditions and the two modules of the *DrinkingLess* intervention that all participants receive. A small-to-medium effect size of *f* = 0.15 was therefore specified, for which the required sample size is 351 (see Figure 7).

### Randomisation

Participants meeting the inclusion criteria will be automatically assigned to one of the 8 intervention conditions with an equal likelihood, using the method of minimisation [55] in order to balance for gender and AUDIT risk level (scores 8–19 versus >19). Participants will be randomly allocated to one of the conditions to which the fewest participants of their gender and AUDIT level have so far been assigned. Intervention allocation will be corrected for excluded participants. Participants will be excluded if (a) they do not complete the baseline assessment including *DrinkingLess*, or (b) if they indicate that they want to discontinue the study.

### Blinding

Outcome assessments are inherently blinded, since they occur online, thus in the absence of assessors and researchers. In order to keep participants blind to their intervention condition, participants are required to respond to an irrelevant feature in all CBM interventions (e.g. the format of the picture) instead of the content of the picture (alcoholic or non-alcoholic beverage). Participants’ awareness of intervention condition is assessed at the post-intervention measurement.

### Clinical evaluation

All analyses will be conducted in agreement with the intention to treat principle, as per the CONSORT statement [56]. To that end, missing endpoints will be imputed using the EM algorithm [57]. The main effects and interaction effects of the CBM interventions on the primary and the continuous secondary outcome measures will be analysed with a 2 (attentional bias retraining: active/placebo) × 2 (alcohol/no-go training: active/placebo) × 2 (approach bias retraining: active/placebo) × 2 (time: pre-intervention/3 month follow-up) repeated measures ANOVA.

### Economic evaluation

The economic evaluation will be conducted from the societal perspective and encompass costs stemming from the intervention, health care uptake and productivity losses due to absenteeism and work cut-back (presenteeism). These costs will be computed from the TiC-P using the full economic cost prices as reported in the pertinent Dutch guideline for assessing health care costs [58]. The costs will be indexed for the reference year 2012. Treatment response will be defined as drinking below the normative cut-off of 21 units per week for men, and 14 units per week for women. The experimental conditions will be compared to the placebo condition to compute the incremental costs and the incremental effects in order to arrive at the incremental cost-effectiveness ratio (ICER). Since the time horizon of this study is less than one year, costs and effects will not be discounted. Employing non-parametric bootstraps, we will simulate 2,500 ICERs and project these on the ICER plane. For medical decision-making purposes, we will also produce the ICER acceptability curve, when the experimental intervention happens to be more effective but also more costly. Sensitivity analyses will be directed at the main cost-drivers with the greatest amount of uncertainty. The same procedure will be used again with quality adjusted life years (QALYs) as the central clinical endpoint. QALYs will be computed from the EQ-5D data while using the Dutch utility tariffs [59].

## Discussion

This trial protocol describes the design of a double-blind randomised controlled trial to assess the effectiveness of a web-based CBM intervention, including attentional bias retraining, alcohol-no/go training, and approach bias retraining in a 2×2×2 factorial design. Since this may prove to be a very cost-effective intervention, an economic evaluation is conducted alongside the trial. To the best of our knowledge, this is the first study to investigate the separate and combined effects of various web-based CBM interventions in the field of addiction. So far, the effects of CBM have mostly been studied in heavy drinking students in the lab, and, to a lesser extent, in alcohol dependent patients in addiction clinics. However, given its low costs, and the relative ease of offering online versions, CBM’s greatest potential may lie in the form of online self-help intervention that can be conducted in the privacy of one’s home. The first strength of this study is therefore that it investigates CBM in the setting in which it is most likely to be implemented, if proven effective. Second, this study combines different CBM interventions in a factorial design, allowing us to study possible incremental effects of combining different versions of CBM, and to explore whether training one bias will have generalised effects on other biases. A third strength of the current study is that it combines weakening automatic appetitive processes with strengthening alternative response options, by training participants towards pictures of non-alcoholic drinks, and by preceding CBM training with a CBT-based intervention, *DrinkingLess*. On theoretical and practical grounds, there are indications that combining CBM with elements of motivational and cognitive behavioural therapy is most likely to be successful [60].

The main strength of this study is also its main limitation: the effectiveness of CBM might be threatened by its web-based delivery. First, the less controlled home environment might pose several distractions, both online (incoming e-mails, other browser windows), and ‘offline’ (television, phone calls), which could confound the RT-based CBM interventions. To this end, participants are reminded at the start of each CBM session of the importance of concentration for the effectiveness of the training. The feasibility of web-based CBM is supported by a recent study on attentional bias retraining in social anxiety, which found error rates and reactions times comparable to those in a lab-based setting, though no evidence was found of training effects [61]. Second, online interventions are commonly faced with high attrition rates [62]. Therefore, treatment adherence is encouraged in five ways: 1) by automatic e-mails that are sent to invite and remind participants of CBM sessions 2) the possibility of e-mail contact with the first author in case participants have questions or encounter technical difficulties 3) the attention that was paid to the intervention rationale 4) the inclusion of motivating features to the CBM interventions 5) a lottery system in which participants who complete the follow-up assessments can win gift vouchers. Taken together, the possible threats presented by the web based setting of this study are outweighed by the possibilities offered by internet-delivered CBM, which, if proven effective, could easily be implemented as a low threshold, early intervention for problem drinkers.

## Abbreviations

AUDIT: Alcohol use disorders identification Test; CBM: Cognitive bias modification; CBT: Cognitive behavioural therapy; TLFB: Timeline-follow-back.

## Competing interests

The authors declare that they have no competing interests.

## Authors’ contributions

DvD constructed the design, is responsible for the data-collection and drafted the manuscript, ES and RW are supervisors, constructed the design and revised the manuscript, RW applied for the grant, FS designed economic evaluation and revised the manuscript, JK developed the *DrinkingLess* intervention and revised the manuscript. All authors read and approved the final manuscript.

## Pre-publication history

The pre-publication history for this paper can be accessed here:

http://www.biomedcentral.com/1471-2458/13/674/prepub
